# Association between Wood and Other Biomass Fuels and Risk of Low Birthweight in Uganda: A Cross-Sectional Analysis of 2016 Uganda Demographic and Health Survey Data

**DOI:** 10.3390/ijerph19074377

**Published:** 2022-04-05

**Authors:** Joshua Epuitai, Katherine E. Woolley, Suzanne E. Bartington, G. Neil Thomas

**Affiliations:** 1 Institute of Applied Health Research, University of Birmingham, Edgbaston, Birmingham B15 2TT, UK; jxe078@alumni.bham.ac.uk (J.E.); kew863@student.bham.ac.uk (K.E.W.); s.bartington@bham.ac.uk (S.E.B.); 2 Department of Nursing, Faculty of Health Sciences, Busitema University, Mbale P.O. Box 1460, Uganda

**Keywords:** biomass cooking fuels, low birthweight, pregnancy outcomes, Uganda, household air pollution

## Abstract

In utero exposure to household air pollution (HAP) from polluting cooking fuels has been linked to adverse pregnancy outcomes including low birthweight (LBW). No previous study in Uganda has attempted to investigate the association between the different types of biomass cooking fuels and LBW. This study was conducted to investigate the association between wood and other biomass cooking fuel use with increased risk of LBW, using the 2016 Uganda Demographic and Health Survey for 15,270 live births within five years prior to interview. LBW, defined as birthweight of <2500 g, was estimated from maternal recall and health cards. Association between household exposure to the different solid biomass cooking fuels and LBW was determined using multivariable logistic regression. Biomass cooking fuels were used in 99.6% of the households, with few (0.3%) using cleaner fuels and 0.1% with no cooking, while the prevalence of LBW was 9.6% of all live-births. Although the crude analysis suggested an association between wood fuel use and LBW compared to other biomass and kerosene fuel use (AOR: 0.82; 95% CI: 0.67–1.00), after adjusting for socio-demographic and obstetric factors, no association was observed (AOR: 0.94; 95% CI: 0.72–1.22). LBW was significantly more likely among female neonates (AOR: 1.32 (95% CI: 1.13–1.55) and neonates born to mothers living in larger households (AOR: 1.03; 95% CI: 1.00–1.07). LBW was significantly less likely among neonates delivered at term (AOR: 0.39; 95% CI: 0.31–0.49), born to women with secondary or tertiary level of education (AOR: 0.80; 95% CI: 0.64–1.00), living in households with a higher wealth index (AOR: 0.69; 95% CI: 0.50–0.96), Eastern (AOR: 0.76; 95% CI:0.59–0.98) and Northern (AOR: 0.75; 95% CI: 0.57–0.99) regions. The study findings suggest inconclusive evidence regarding the association between the use of wood compared to other biomass and kerosene cooking fuels and risk of LBW. Given the close observed association between socioeconomic status and LBW, the Ugandan government should prioritize public health actions which support female education and broader sustainable development to improve household living standards in this setting.

## 1. Introduction

Globally, about 2.8 billion (36%) people rely on kerosene and biomass fuels (charcoal, coal, wood, animal dung, and agricultural residues), referred to collectively as biomass fuels, for cooking and heating [1,2]. In Asia, significant progress in transitioning to cleaner fuels (liquid petroleum gas [LPG], electricity) has been seen, but little progress has been made in Africa, where the proportion of solid fuel use has only decreased slightly from 90% in 1990 to 84% in 2020 [1]. The limited decrease in proportion of biomass cooking fuel use in Africa is offset by an absolute increase in the population that solely relies on polluting cooking fuels due to population growth [1]. By 2025, the use of biomass fuels for cooking is projected to exceed one billion people in Africa [1]. Biomass fuels often undergo incomplete combustion, producing high levels of household air pollution (HAP), which includes particulate matter (PM) and carbon monoxide (CO) at levels that frequently exceed the World Health Organization Global Air Quality Guidelines (WHO-AQGs) [2].

Globally, exposure to HAP results in approximately four million deaths annually, 4.8% of all disability-adjusted life years, and 7.7% of global mortality [3], and is associated with increased risk of upper and lower respiratory tract infections, asthma, cataracts, lung cancer, chronic obstructive pulmonary disease, tuberculosis, and cardiovascular diseases [3,4]. In addition, women and girls are predisposed to musculoskeletal injuries, sexual violence, animal bites, insect-borne disease, and widening of gender inequities related to the gathering of biomass fuels [5].

Women and children under five years of age are more vulnerable to the adverse effects of HAP, due to gender-patterned cooking roles which lead to greater HAP exposure among females, while children aged under five years are often kept in close proximity to their mothers during cooking periods [6]. Exposure to HAP in utero, a particularly vulnerable stage of fetal development [6], has been associated with increased risk of adverse pregnancy outcomes including increased risk of low birthweight (LBW), preterm birth, small for gestational age, spontaneous abortion, stillbirth, and neonatal mortality [4,7,8]. Globally, 90% of 4 million neonatal deaths annually occur in developing countries, including Uganda [9]. LBW prevalence is disproportionately high (14%) in Sub-Saharan Africa [10], and is one of the leading causes of neonatal morbidity and mortality in Sub-Saharan Africa [9].

Studies and meta-analyses have identified an association between exposure to biomass compared to cleaner fuels (LPG, electricity) with increased risk of LBW [4,7,11,12]. However, only a limited number of studies from Africa were included in the meta-analyses [4,7,11,12], a region with a high burden of LBW [10], and predominant use of polluting fuels [1]. The studies from Africa were limited to relatively few countries, including Ghana [13], Nigeria [14,15,16,17], Malawi [18], Ethiopia [19,20,21], and Zimbabwe [22]. The African studies were limited to hospital-based settings [21] with a reliance on the woman’s subjective recall of the birth weight or size of the child [14,18,22]. The studies did not address the effect of outdoor or indoor place of cooking on LBW risk [13,14,15,17,23], or generally control for important confounders [18]. Moreover, there remains limited understanding of the relationship between different biomass fuel types and risk of LBW, which is of importance in Sub-Saharan Africa where access to cleaner fuels remains limited. In Uganda, the only two studies focusing on HAP that were included in the review were limited to respiratory symptoms only [24,25,26]. To the best of our knowledge, no study in Uganda has investigated the impact of different types of biomass fuels on the risk of LBW.

Our study was conducted in Uganda, a low-income country in Sub-Saharan Africa [27], where 97% of the households predominantly use biomass fuels for cooking [28]. Uganda, with a population of about 40 million people, has a gross domestic product (GDP) per capita of 958.19 USD [27]. The maternal mortality rate is 336 per 100,000 live births with a neonatal mortality rate of 27 per 1000 live births [28]. The high gender inequality in Uganda means that household activities such as cooking and collecting firewood are usually undertaken by women [29]. About 21% of the population in Uganda live below the poverty line, with poor housing and under-ventilated living conditions experienced by over half (56%) of its population. Although at a national level ambitious energy policy targets have been set to promote access and adoption of cleaner fuels by 2040, the lack of innovative implementation strategies to scale up adoption of cleaner fuel alternatives may have greatly restricted real-world realization of these policy goals [30].

The low proportion of households using cleaner fuels, corresponding high use of wood fuel, and relatively high utility of switching from raw to processed biomass fuels (e.g., wood to charcoal) suggests the need to investigate the relative risk of LBW if households used other biomass fuels rather than wood. The aim of this study was therefore to determine the association between the use of wood compared to other types of biomass and kerosene cooking fuels and the risk of LBW in Uganda, using the population-based 2016 Uganda Demographic and Health Survey (UDHS).

## 2. Materials and Methods

### 2.1. Study Design, Setting, and Data Source

The UDHS (2016) is a cross-sectional population-based national dataset funded by the U.S. Agency for International Development, with the birth recode (a file produced by DHS where each observation is an individual birth within the last five years) and relevant variables from the individual recode being extracted for this study [28]. The birth recodes contained birth history data, while individual recode (each observation is every woman within the survey) provided information on socio-demographic and household characteristics [28]. A two-stage stratified sampling methodology was employed to randomly select a representative sample [28]. Any woman residing in the selected household of reproductive age (15–49 years) was interviewed and asked to report their birth history (including live and still births) for the five years preceding the survey. A total of 19,588 households and 18,506 women were surveyed [28], with response rates of 97% (18,506/19,088) for the individual (women’s) dataset and 67% (10,429/15,522) of the birth records respectively. Additional information regarding the UDHS has been described elsewhere [28].

### 2.2. Study Population

Singleton live births, occurring at term (≥37 weeks gestation) and/or pre-term (<37 weeks gestation) which occurred in the last five years (2012–2016) from the time of interview were included in the study. Multiple births were excluded from the analysis because of the high risk of LBW among multiple pregnancies [31].

### 2.3. Modifications to the Wealth Index

The wealth index provided by DHS is calculated through principal component analysis (PCA), including assets, toilet facility, drinking water sources, cooking fuel, and house construction as predictor variables [32], with the final variable containing wealth quintiles (lowest, low, middle, high, and highest). As cooking fuel was the exposure of interest within this study, the wealth index (categorized as low, second, middle, fourth, and higher) was recalculated using the methods provided by the DHS [33] in SPSS [34] to remove cooking fuel to prevent circularity [35].

### 2.4. Exposure Variables

Self-reported main household cooking fuel was categorized into cleaner fuels (LPG), electricity, biogas, and no cooking), and biomass and kerosene fuels (kerosene, coal/lignite, charcoal, wood, straw/shrubs/grass, agricultural crop, animal dung). Households, where cooking was not done, were classified as using cleaner fuel because it was assumed that HAP levels in these households would be comparable to households that used cleaner fuels. The exposure variable was the biomass or kerosene cooking fuels, categorized into wood and other biomass and kerosene fuels (kerosene, charcoal, straw/shrubs/grass, agricultural crop, animal dung).

### 2.5. Outcome Variables

The outcome variable was LBW defined as a birthweight of less than 2500 g, obtained from either the health card (34%) or the maternal recall of child’s weight at birth (66%).

### 2.6. Covariates

Covariates were identified from the literature [21,36,37,38] as those potentially associated with HAP or LBW. The covariates from household and contextual characteristics included age of the household head, access to electricity, place of residence (urban, rural), geographical region (central, east, north, west), household smoking status (yes, no), place of cooking (in the main house, separate house, outdoors) and wealth index (low, second, middle, fourth, or highest). The 15 sub-regions in Uganda were categorized into four regions which included central, east, west, and north, which are defined based on ethnicity, poverty index, and geographical location [39]. Information from the respondents included age (15–19, 20–34, 35–49 years), level of education (no or primary education, secondary or tertiary). Pregnancy-related maternal covariates considered included parity (primigravida or multigravida), birth order (continuous variable), sex of the baby (male or female), and body mass index (BMI) (<18.5 or ≥18.5 kg/m^2^). Circumstantial pregnancy-related covariates included mode of delivery (spontaneous vaginal delivery or caesarian section), the timing of first antenatal care visit (ANC) (≤5 or >5 months), number of ANC visits (≥4 or <4), sulphadoxine-pyrimethamine (SP) (yes or no), birth interval (<24 months or ≥24 months), iron-folate supplementation (yes or no), deworming during pregnancy (yes or no), birth interval, based on WHO categorization, of less than 24 months or birth intervals of ≥24 months was used [40]. BMI, measured in kg/m^2^, was categorized as low when BMI was <18.5 or normal when BMI was ≥18.5 [41], as there is a higher risk of LBW for BMI of less than 18.5 [42,43].

### 2.7. Data Analysis

Categorical variables were summarized using frequencies and proportions. Skewed continuous variables were summarized using the median and inter-quartile range (IQR), while normally distributed continuous variables were summarized using means and standard deviations. Bivariate and multivariate logistic regression, using survey commands to adjust for the complex sampling structure, was deployed to determine the association between exposure to wood and other forms of polluting fuels and LBW. The odds ratios (OR), 95% confidence intervals (95% CI), and *p*-values were reported. Clinically relevant variables, those with a *p*-value was less than 0.2, and variables without high levels of missing, in the bivariate analyses were included in the multivariate logistic regression model. Missing values were handled by case-wise deletion.

**Figure 1:**
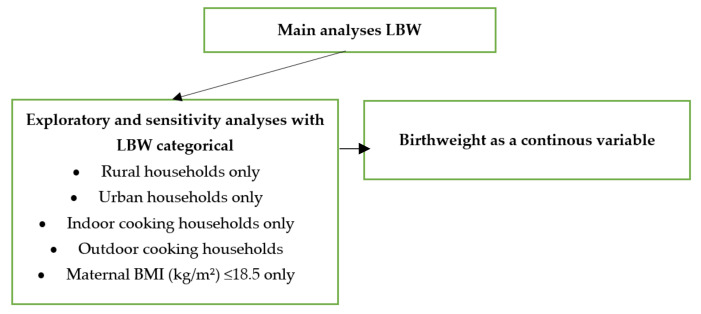
Description of sensitivity analysis.

Sensitivity analyses were undertaken to ensure robustness of study findings and to further investigate confounding factors (e.g., BMI) that could not be accounted for in the main analysis due to a large proportion of missing data (Figure 1). Further stratified analyses were undertaken according to residence (rural or urban), cooking location (indoor, outdoor), and maternal BMI (≤18.5). Multivariable linear regression model was performed using birthweight as a continuous variable. Stata software (version 16.1) [44] was used to analyze the data.

### 2.8. Ethical Approval and Authorisation

USAID obtained ethical approvals from the relevant authorities in Uganda to collect the data. Permission was obtained from the USAID to gain access to the anonymized and aggregated freely available dataset from the DHS online data archive [45].

## 3. Results

### 3.1. Descriptive Statistics

**Figure 2:**
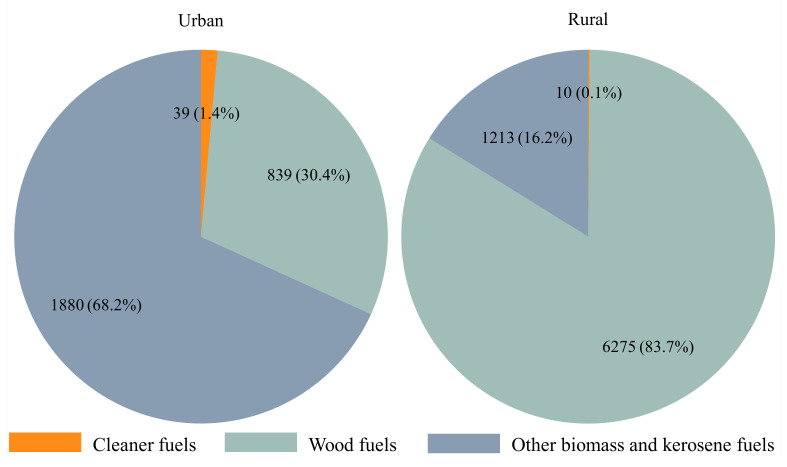
Description of cooking fuel use in rural and urban settings. Other biomass and kerosene = kerosene, charcoal, straw/shrubs/grass, agricultural crop, animal dung.

Out of 15,270 births recorded in the last five years, birthweights were available for 10,267 births (67.2%), with 986 births (9.6%) classified as LBW (Table 1). Of these births, cleaner fuels (LPG, biogas & electricity) were used in 0.3% (*n* = 48) households, while biomass fuels and kerosene were used in 99.6% (*n* = 15,209) of the households. Overall 11% (*n* = 2168) of the household respondents reported cooking to be performed inside the main residential house, of which 68% (*n* = 1478) did not have a separate room as a kitchen. The dominant household cooking fuel was mostly charcoal in urban areas (*n* = 1880, 68.2%) and wood in rural areas (N = 6272, 83.7%) (Figure 2). Cleaner fuels (LPG, electricity) were only observed for 39 births (1.4%) in urban and 10 births (0.1%) in rural areas. Risk of LBW was significantly associated with female sex (56% vs. 49%), pre-term delivery (28% vs. 13%), less than four antenatal visits (41% vs. 34%), a lower prevalence of sulphadoxine-pyrimethamine (77% vs. 81%) or deworming treatment (60% vs. 65%) (Table 2). 

### 3.2. Association between Type of Biomass Cooking Fuels with LBW

In the bivariate analysis, a borderline association was observed between LBW in households using wood cooking compared to other biomass fuels (OR: 0.82; 95% CI: 0.67–1.00) (Table 3). However, after adjustment for socio-demographic and obstetric factors, the association was no longer significant (AOR: 0.94; 95% CI: 0.72–1.22). A number of the covariates were associated with LBW risk, including the size of household, where for every additional household member the odds ratio of LBW increased by 3% (AOR: 1.03; 95% CI: 1.00–1.07). Having a higher level of maternal education was associated with lower odds ratio of LBW in other polluting fuel-cooking (AOR: 0.80; 95% CI: 0.64–1.00) households compared to wood-cooking households, as was being female (AOR: 1.32; 95% CI: 1.13–1.55) compared to being male. Pregnancies that lasted longer than 37 weeks were also associated with lower odds of LBW (AOR: 0.39; 95% CI: 0.31–0.49) compared to pregnancies that were less than 37 weeks gestation. Likewise, the fourth highest wealth index was associated with lower odds of LBW compared to the lowest wealth index (AOR: 0.69; 95% CI: 0.5–0.96)

### 3.3. Sensitivity Analyses

Place of residence, cooking location, and adjustment for maternal BMI in sensitivity analyses all had no observed association of LBW between births from wood cooking compared to other biomass cooking households (Table 4). Birthweight was explored as a continuous variable, with births from other polluting fuels-cooking households having a 29.43 g higher (95% CI: −38.15, 76.01) birthweight compared to wood cooking, however, this was not statistically significant (*p* = 0.393). In the sensitivity analyses, which included the addition of all available confounders including mothers BMI, the timing of ANC visit, and number of ANC visits, no significant association (AOR = 0.78 (95% CI: 0.46, 1.31) was found among households where wood compared to other biomass fuels were used.

## 4. Discussion

This population-based study of 15,270 live-births and a high response rate (97%) in Uganda was conducted to determine the association between wood compared to kerosene and other biomass cooking fuels with the risk of LBW. No evidence of an association was observed between the different types of biomass and kerosene fuels and risk of LBW (AOR: 0.94; 95% CI: 0.72, 1.22) after adjustment for confounding factors. In the sensitivity analyses which considered the associated effect of residence (rural, urban), cooking location (indoor, outdoor), maternal BMI, and birthweight as a continuous dependent variable with risk of LBW, findings similar to the main analyses were found.

LBW was observed to occur among almost 10% of all deliveries which, although lower than the 14% previous estimates for Sub-Saharan Africa [10], reflects a major public health concern. LBW, which may partly result from HAP exposure, is one of the leading causes of neonatal mortality in developing countries [9], which therefore calls for further research, innovations, and policies to reduce the health burden of LBW brought by use of biomass cooking fuels.

To the best of our knowledge, this was the first study in Uganda to determine the association between the different types of biomass fuels and LBW, a setting where biomass fuels were used by almost all (99.6%) of the population; limiting the possibility of comparing biomass fuels to cleaner fuels. The DHS collects robust, nationally representative data, however, there were a large (32.8%) number of missing birthweights recorded. However, 67.2% of birthweights were recorded totaling 15,270 live-births. Birthweights obtained by maternal recall may have been subject to recall bias, but this was reduced by limiting the data collection period to births within the last five years. Although there was no evidence of an effect in our study and the reference cooking fuel differs to that of other studies, they are similar to those obtained in single-country analyses investigating the difference between biomass and cleaner fuels in Malawi (AOR, 1.29 (95% CI: 0.34; 4.48) [18]), Ethiopia (AOR, 1.3 (95% 0.9–1.9) [20]), Nigeria (−0.09 (95% CI: −0.31, 0.10) [17]), Bangladesh (AOR 0.96 (95% CI: 0.81–1.13) [46]), and India (AOR: 1.07(95% CI: 0.94–1.22) [47]). On the contrary, findings in this study were inconsistent with the population-based study from Zimbabwe which showed biomass fuel use was associated with a 175 g lower birthweight (95% CI: −300 to −50 g) [22]. While solid biomass fuels significantly increased the risk of LBW by 35% in a meta-analysis of 19 studies, the association was not significant for a sub-group analysis of studies from Africa [7]. Similarly, a systematic review of DHS studies found that two studies out of five from African countries (Malawi and Zimbabwe) cited no evidence of an association between biomass cooking fuels and LBW, which was consistent with findings in this study [48]. In African settings like Uganda where biomass cooking fuels are almost universally used and the consequent small proportion that use cleaner fuels, direct measures of exposure to HAP may be more suited to investigate the effect of HAP on maternal and child health.

There is evidence for the biological plausibility of LBW risk associated with in-utero HAP exposure including PM_2.5_, CO, and aromatic hydrocarbons [12,49]. Increased risk of LBW has been observed among infants exposed to second-hand smoke and HAP exposure in animals [7,12,50]. Exposure to polluting fuels is proposed to cause LBW by reducing fetal growth [7,49]. Reduced fetal growth occurs through impaired maternal lung function, fetal toxicity, endocrine disruption, pathological placental changes, oxidative processes, and reduced oxygen and micronutrients supply to the fetus [7,12,16,49,51]. The difference in pollutant levels between wood and other biomass may not differ or reduce enough to produce an effect, along with households often switching between biomass fuel types. In addition, the WHO has stated in the updated AQGs that there is no safe level of PM [2] and levels of CO remain high [2]; therefore, barriers should be broken down to allow access to cleaner alternatives.

Charcoal was predominantly used in urban settings, and wood was mostly used in rural settings, but no difference in odds of LBW risk was observed between rural and urban areas. In settings where biomass fuels are mostly used and access to cleaner fuels remains limited, feasible mitigation measures should be promoted to reduce exposure to HAP among vulnerable women and children under five years of age [24]. The mitigation measures such as cooking outdoors, keeping children away from cooking places, opening doors and windows, improving housing and ventilation conditions, use of cured wood or charcoal, and building kitchens with chimneys might play a vital role in modifying fuel user behaviors, the environment, and the source of HAP [5,52]. These addressable mitigation measures may not only reduce exposure to HAP but would also create awareness and facilitate uptake of long-term active measures such as scaling up the adoption of cleaner fuels [53]. In the long term, transitioning to cleaner fuels should be prioritized as the ultimate solution to reducing the impact of HAP.

Although improved cookstoves can still emit levels of PM_2.5_ and CO which exceed WHO-AQGs [54], a reduced risk of LBW has been observed with the use of improved cookstoves compared to traditional stoves in a systematic review and meta-analysis [55]. Government can prioritize the adoption of improved cookstoves as the first step in transitioning to cleaner fuels [5]. The promotion of cleaner fuels requires government commitment, strong energy fuel policies, subsidies, and tax exemptions on modern fuels [5]. Programs should prioritize pregnant women to reduce the adverse effects of HAP on pregnancy [56].

Proxy measures may be reliable and valid ways of estimating HAP exposure, however, the inability to use direct measures of HAP may have resulted in misclassification bias and inaccurate estimation of HAP. Further DHS data collection should endeavor to check birthweight against health card and capture use of secondary cooking fuels, stove type, alongside fuel and stove stacking practices [47]. Future studies should ideally use a direct measurement of personal exposure to HAP to investigate the association between biomass fuels and LBW. Direct measures of HAP would help determine an exposure-response relationship. Causality could not be determined in this study, therefore, robust study designs such as prospective cohort studies or if feasible, randomized controlled trials should be used to infer causality. We recommend exploration of other sources of HAP on LBW risk, including outdoor burning of garbage in the household including burning of solid biomass fuels and wastes from the roadsides and use of polluting fuels for lighting.

## 5. Conclusions

In this study, almost all (99.6%) study households in Uganda used biomass and kerosene fuels for domestic cooking, with LBW observed among 9.6% of singleton live births. After adjusting for household, maternal and demographic confounding factors, no evidence of an association was observed between risk of LBW and the use of wood fuel compared to other biomass or kerosene cooking fuels. Although previous evidence has indicated reduced risks of LBW with cleaner fuels, this requires further investigation, as the very low proportion of LPG, electricity or biogas use meant a comparison of risks associated with biomass fuels could not be undertaken in this present study. Achieving energy policy targets for adoption of cleaner fuels by 2040 in Uganda remains a key policy priority given the existing high reliance upon polluting biomass fuels. Public health initiatives to reduce LBW prevalence should seek to reduce socio-economic inequity and increase levels of female education in this context.

## Figures and Tables

**Table 1:** Household characteristics by low birthweight classification of study participants.

	Birthweight < 2500 g N (%)	Birthweight ≥ 2500 g N (%)	
	N = 986	N = 9281	*p*-Value
**Cooking fuel**			0.062
Wood	720 (73.3)	6393 (69.3)	
Other polluting fuels	262 (26.7)	2831 (30.7)	
Missing (%)	4 (0.4)	56 (0.6)	
**Cooking location**			0.942
In the house	100 (10.2)	967 (10.4)	
In a separate building	622 (63.1)	5774 (62.3)	
Outdoors	264 (26.8)	2521 (27.2)	
Missing (%)	264 (26.8)	0 (0.0)	
**Household smoking**			0.863
Yes	862 (87.5)	8137 (87.7)	
No	124 (12.5)	1144 (12.3)	
**Type of place of residence**			0.293
Urban	247 (25.0)	2522 (27.2)	
Rural	739 (75.0)	6759 (72.8)	
**Region**			0.096
Central	277 (28.1)	2719 (29.3)	
East	250 (25.3)	2354 (25.4)	
North	259 (26.3)	2071 (22.3)	
West	200 (20.3)	2137 (23.0)	
**Electricity**			0.011
Yes	700 (74.3)	6169 (69.0)	
No	243 (25.7)	2772 (31.0)	
Missing (%)	43 (4.4)	339 (3.7)	
**Number of household members (listed)**			0.568
Median (IQR)	6.0 (4.0, 8.0)	5.0 (4.0, 7.0)	
**Combined wealth index**			0.003
Low	248 (25.1)	1851 (19.9)	
Second	193 (19.6)	1662 (17.9)	
Middle	182 (18.4)	1632 (17.6)	
Fourth	158 (16.1)	1804 (19.4)	
Highest	205 (20.8)	2332 (25.1)	
**Maternal characteristics**			
**Respondent’s current age**			<0.001
Median (IQR)	26.0 (22.0, 32.0)	27.0 (23.0, 32.0)	
**Mother’s education**			0.010
No education/Primary	697 (70.7)	6072 (65.4)	
Secondary/higher	289 (29.3)	3208 (34.6)	
**Mother’s BMI (kg/m^2^)**			0.223
<18.5	19 (6.2)	253 (8.3)	
≥18.5	296 (93.8)	2795 (91.7)	
Missing (%)	670 (68.0)	6233 (67.2)	

Footnotes: IQR = Inter quartile range.

**Table 2:** Birth characteristics by low birthweight classification of study participants.

Variable	Birthweight < 2500 g N (%)	Birthweight ≥ 2500 g N (%)	
	N = 986	N = 9281	*p*-Value
**Parity**			0.327
Once	170 (17.2)	1466 (15.8)	
More than once	816 (82.8)	7814 (84.2)	
**Birth order number**			
Median (IQR)	2.0 (1.0, 5.0)	3.0 (2.0, 5.0)	
**Sex of child**			<0.001
Male	438 (44.4)	4761 (51.3)	
Female	548 (55.6)	4520 (48.7)	
**Birth Interval**			0.576
≤24 months	824 (83.5)	7681 (82.8)	
>24 months	162 (16.5)	1599 (17.2)	
**Duration of pregnancy**			<0.001
Pre-term	274 (27.8)	1184 (12.8)	
Term	711 (72.2)	8097 (87.2)	
**Timing of first ANC visits**			0.066
<5 months gestation	387 (65.1)	3896 (60.8)	
≥5 months gestation	207 (34.9)	2509 (39.2)	
Missing (%)	391 (39.7)	2875 (31.0)	
**Number of ANC visits**			0.003
<4	252 (41.4)	2206 (34.3)	
≥4	357 (58.6)	4225 (65.7)	
Missing (%)	377 (38.3)	2849 (30.7)	
**Place of delivery**			0.131
Health facility	906 (92.5)	8655 (93.9)	
Home	73 (7.5)	561 (6.1)	
Missing (%)	7 (0.7)	64 (0.7)	
**Delivery by caesarean section**			0.763
No	895 (91.4)	8479 (91.8)	
Yes	84 (8.6)	760 (8.2)	
Missing (%)	7 (0.7)	42 (0.4)	
**Iron supplementation**			0.131
No	66 (10.8)	562 (8.7)	
Yes	545 (89.2)	5906 (91.3)	
Missing (%)	375 (38.0)	2812 (30.3)	
**Sulphadoxine-pyrimethamine**			0.014
Yes	467 (76.8)	5251 (81.4)	
No	141 (23.2)	1199 (18.6)	
Missing (%)	378 (38.4)	2831 (30.5)	
**Deworming**			0.021
Yes	364 (59.7)	4179 (65.2)	
No	246 (40.3)	2226 (34.8)	
Missing (%)	376 (38.1)	2876 (31.0)	

**Table 3:** Unadjusted and adjusted analysis for the association between LBW and type of biomass cooking fuel.

	Unadjusted Analysis			Adjusted Analysis (N = 9863)		
	UOR	95% CI	*p*-Value	AOR	95% CI	*p*-Value
**Household Characteristics**						
**Cooking fuel**						
Wood	Ref.			Ref.		
Other polluting fuels	0.82	(0.67, 1.00)	0.053	0.94	(0.72, 1.22)	0.646
**Cooking location**						
In the house	Ref.			Ref.		
In a separate building	1.04	(0.75, 1.43)	0.818	0.99	(0.71, 1.36)	0.928
Outdoors	1.01	(0.71, 1.43)	0.959	0.94	(0.67, 1.30)	0.691
**Household smoking**						
Yes	Ref.			Ref.		
No	1.02	(0.82, 1.27)	0.863	0.92	(0.73, 1.15)	0.470
**Type of place of residence**						
Urban	Ref.					
Rural	1.12	(0.91, 1.37)	0.294	0.90	(0.70, 1.15)	0.400
**Region**						
Central	Ref.			Ref.		
East	1.04	(0.83, 1.30)	0.726	0.76	(0.59, 0.98)	0.035
North	1.23	(0.98, 1.54)	0.076	0.75	(0.57, 0.99)	0.042
West	0.92	(0.71, 1.19)	0.525	0.82	(0.62, 1.06)	0.134
**Electricity**						
Yes	Ref.			Ref.		
No	1.30	(1.06, 1.58)	0.011	0.94	(0.73, 1.22)	0.655
**Number of household members (listed)**						
	1.01	(0.99, 1.04)	0.418	1.03	(1.00, 1.07)	0.027
**Combined wealth index**						
Low	Ref.			Ref.		
Second	0.87	(0.70, 1.08)	0.214	0.92	(0.71, 1.18)	0.500
Middle	0.83	(0.66, 1.04)	0.108	0.89	(0.67, 1.19)	0.435
Fourth	0.66	(0.51, 0.84)	0.001	0.69	(0.50, 0.96)	0.027
Highest	0.66	(0.50, 0.85)	0.002	0.73	(0.50, 1.08)	0.120
**Maternal characteristics**						
**Respondent’s current age**						
	0.98	(0.97, 0.99)	0.001	0.99	(0.97, 1.01)	0.403
**Mother’s education**						
No education/Primary only	Ref.			Ref.		
Secondary only/higher	0.78	(0.65, 0.94)	0.010	0.80	(0.64, 1.00)	0.050
**Mother’s BMI (Kg/m^2^)**						
<18.5	Ref.					
≥18.5	1.37	(0.82, 2.29)	0.225			
**Birth characteristics**						
**Parity**						
Once	Ref.			Ref.		
More than once	0.90	(0.73, 1.11)	0.327	1.18	(0.89, 1.55)	0.249
**Birth order number**						
	0.95	(0.92, 0.99)	0.008	0.96	(0.90, 1.02)	0.164
**Sex of child**						
Male	Ref.			Ref.		
Female	1.32	(1.13, 1.53)	<0.001	1.32	(1.13, 1.55)	0.001
**Birth Interval**						
≤24 months	Ref.			Ref.		
>24 months	0.95	(0.78, 1.15)	0.576	0.95	(0.77, 1.16)	0.597
**Duration of pregnancy**						
Pre-term	Ref.			Ref.		
term	0.38	(0.31, 0.46)	<0.001	0.39	(0.31, 0.49)	<0.001
**Timing of first ANC visits**						
<5 months gestation	Ref.					
≥5 months gestation	0.83	(0.68, 1.01)	0.066			
**Number of ANC visits**						
<4 times	Ref.					
≥4 times	0.74	(0.61, 0.90)	0.003			
**Place of delivery**						
Health facility	Ref.			Ref.		
Home	1.25	(0.94, 1.67)	0.131	1.21	(0.90, 1.62)	0.211
**Delivery by caesarean section**						
No	Ref.			Ref.		
Yes	1.05	(0.78, 1.41)	0.763	1.16	(0.87, 1.56)	0.313
**Iron supplementation**						
No	Ref.					
Yes	0.78	(0.57, 1.08)	0.132			
**Sulphadoxine-pyrimethamine**						
Yes	Ref.					
No	1.32	(1.06, 1.65)	0.014			
**Deworming**						
Yes	Ref.					
No	1.27	(1.04, 1.55)	0.021			

Footnotes: UOR = unadjusted odds ratio, AOR = adjusted odds ratio, 95% CI = 95% confidence interval, Ref = reference group.

**Table 4:** Exploratory and sensitivity analysis.

	AOR	95% CI	*p*-Value
**Place of residence**			
Urban (N = 2274)	1.09	(0.65, 1.83)	0.745
Rural (N = 7589)	0.85	(0.61, 1.18)	0.322
**Cooking location**			
Indoor (N = 7190)	1.04	(0.77, 1.42)	0.786
Outdoor (N = 2682)	0.77	(0.49, 1.19)	0.235
**Adjusting for normal maternal BMI**			
BMI ≤ 18 (N = 2655)	0.92	(0.59, 1.44)	0.726
**Adjusting for all confounders**			
N = 2167	0.78	(0.46, 1.31)	0.348
**Birthweight as a continuous variable**			
N = 9863	29.43	(−38.15, 97.01)	0.393

Footnotes: AOR = adjusted odds ratio, 95% CI = 95% confidence interval. Adjusted for cooking location, household smoking, type of place of residence, region, electricity, wealth index, respondents current age, mother’s education, mothers BMI, parity, birth order, sex of child, birth interval, duration of pregnancy, the timing of ANC visit, number of ANC visits, place of delivery and mode of delivery.

## Data Availability

The data presented in this study are available on request from the corresponding author.
